# Diagnostic Use of CCR3, CD63, CD203c and FcεRIα on Blood Leukocytes of Allergic Asthma and Combined Allergic Rhinitis and Asthma Syndrome

**DOI:** 10.1111/jcmm.70594

**Published:** 2025-06-18

**Authors:** Junling Wang, Mengmeng Zhan, Fangqiu Gu, Huiyun Zhang, Hua Xie, Bingyu Qin, Shaoheng He

**Affiliations:** ^1^ Department of Critical Care Medicine, Henan Provincial People's Hospital Zhengzhou University People's Hospital Zhengzhou Henan China; ^2^ Allergy and Clinical Immunology Research Centre The First Affiliated Hospital of Jinzhou Medical University Jinzhou Liaoning China; ^3^ Translational Medicine Institute Shenyang Medical College Shenyang Liaoning China; ^4^ The PLA Center of Respiratory and Allergic Disease Diagnosing Management General Hospital of Shenyang Military Area Command Shenyang Liaoning China

**Keywords:** allergic asthma, basophil, CCR3, CD203c, combined allergic rhinitis and asthma, eosniophil, granulocyte

## Abstract

Altered basophil identification markers have been discovered to associate with allergic asthma (AA) recently. However, little is known of the expressions of basophil markers in blood granulocytes. We therefore parallel tested them in peripheral blood mononuclear cell (PBMC) and granulocyte populations of patients with AA and combined allergic rhinitis and asthma (ARA) by flow cytometry and assessed their diagnostic performance and examined plasma levels of EDN, LTC_4_ and PGD_2_. We found CCR3^+^ cell numbers increased markedly in granulocytes of AA and ARA patients. Almost all isolated CCR3^+^ PBMC were basophils, but CCR3^+^ granulocytes contained up to 96.0% eosinophils and 12.8% basophils. The numbers of CD63, CD203c and FcεRIα expressing CCR3^+^ granulocytes increased markedly, and the mean fluorescent intensity of CD203c, FcεRIα and CD63 expression on CCR3^+^ granulocytes and PBMC enhanced consistently in AA and ARA patients. The area under the curve achieved the highest values for CD203c^+^CCR3^+^ granulocyte numbers in AA and ARA patients. Plasma levels of EDN, LTC_4_ and PGD_2_ in AA and ARA patients increased and correlated well with CCR3^+^ granulocyte numbers. In conclusion, discovering basophils in peripheral blood granulocytes provides greater scope for the clinical use of basophil tests.

## 1 Introduction

Allergic asthma (AA) is a major phenotype of asthma, which often commences in childhood and is associated with a family history of allergic disease [1]. Actually, AA often coexists with allergic rhinitis and is termed combined allergic rhinitis and asthma syndrome (ARA) [2]. Basophils are known as one of the primary effector cells of allergy [3], which greatly influence the behaviour of eosinophils [4] and neutrophils [5]. As an increased number of basophils in the sputum has been observed in patients with AA [6], this cell type is likely to play a role in AA.

Basophils are easy to obtain from peripheral blood and, therefore, basophil‐based tests should be practicable for allergy diagnosis in most medical labs. The strategy by which basophils are captured in a flow cytometry protocol has met many recommendations and experimental novelties in recent years [7]. Generally, CCR3 has been recognised as a classical surface identification marker of basophils [8], and CD203c and CD63 are considered as activation markers of basophils [9]. It is noteworthy that human peripheral blood eosinophils also express CCR3 [10]. Meanwhile, it is reported that anti‐IgE combined with anti‐CCR3 or anti‐CD203c could achieve very high purity of basophils in most cases [11].

In AA, different results have been produced from various laboratories in recent years. For example, the controversial CCR3^+^ basophils in allergic individuals [12, 13], and increased CD203c^+^ basophils were observed in the blood from patients with asthma exacerbation [14]. To further clarify expressions of CCR3, CD203c, CD63 and FcεRIα in human peripheral blood mononuclear cells (PBMC) and granulocytes, we investigated them herein.

Till now, studies on flow cytometry examination of expression markers of basophils focus on basophils in PBMC and completely ignore basophils in granulocytes. As some of the basophils are multi‐nucleated cells and they were originally discovered as one of the granulocytes in mammals [15], we attempted to parallel examine basophils in PBMC and granulocyte populations in the current study.

Proinflammatory mediator prostaglandin D2 (PGD_2_) is a key mediator of AA [16] that is mainly synthesised by mast cells, eosinophils [17] and basophils [18]. Like PGD_2_, leukotriene C4 (LTC_4_) is also a product of eosinophils [19] and basophils [20], which play an important role in AA [21]. Eosinophil‐derived neurotoxin (EDN) is a major protein content of eosinophil granules but not basophils [22], which is proven to be involved in AA [23]. It is likely that these basophil and eosinophil released products may assist in the diagnosis of AA and distinguish basophil degranulation from eosinophil degranulation.

The aim of the present study is to evaluate the diagnosing value of CCR3, CD203c, CD63 and FcεRIα expression in PBMC and granulocytes of patients with AA and ARA, which may helpfully complete conventional tests, allowing differentiation between allergic and non‐allergic individuals. We also examined the plasma levels of the specific proinflammation mediators of basophil and eosinophil PGD_2_ and LTC_4_, and of eosinophil EDN in patients with AA and ARA, and explored further their correlations with the numbers of CCR3^+^ granulocytes and PBMC.

## 2 Materials and Methods

### 2.1 Reagents

The following reagents were purchased from Biolegend (San Diego, USA): APC‐conjugated mouse anti‐human CCR3 monoclonal antibody (mAb, Clone: 5E8) and its isotype Ab APC‐conjugated mouse IgG2b κ (Clone: MPC‐11), PerCP‐conjugated mouse anti‐human FcεRIα mAb (Clone: AER‐37) and its isotype Ab PerCP‐conjugated mouse IgG2b κ (Clone: MPC‐11), PE‐conjugated mouse anti‐human CD203c mAb (Clone: NP4D6) and its isotype Ab PE‐conjugated mouse IgG1 κ (Clone: MOPC‐21), PE/Cy7‐conjugated mouse anti‐human CD63 mAb (Clone: H5C6) and its isotype Ab PE/Cy7‐conjugated mouse IgG1 κ (Clone: MOPC‐21), Zombie Aqua Fixable Viability Kit, human Fc receptor blocking solution and human erythrocyte lysis buffer. Wright‐Giemsa Stain was supplied by Baso diagnostics Inc. (Zhuhai, China). Allergens for skin prick tests were supplied by ALK‐Abelló Inc. (Denmark). Human PGD_2_ and LTC_4_ ELISA kits were purchased from Cayman Chemical (Ann Arbor MI, USA). Human EDN ELISA kit was obtained from ELISAGenie (Dublin, Ireland). Most of the general‐purpose chemicals, such as salts and buffer components, were of analytical grade.

### 2.2 Subjects

As presented in Table S1, a total of 125 AA patients, 98 ARA patients and 103 healthy control (HC) subjects were included in the study. AA diagnosis, based on the history of characteristic symptom patterns and evidence of variable expiratory airflow limitation, was in accordance with the criteria of the Global Initiative for Asthma [24]. ARA was diagnosed based on allergic rhinitis and its impact on asthma [2]. The mild and moderate AA and ARA patients with physician‐diagnosed atopic history (e.g., eczema, or food or drug allergy) [25] but without other inflammatory conditions or history of taking any anti‐allergy preparation (e.g., antihistamine drugs, steroids, antileukotriene agents, anti‐IgE biologics, anti‐cytokine receptor biologics, or allergen immunotherapy) for at least 2 weeks were recruited in this study. HC subjects in the current study were negative for common airborne and food allergen tests and had no atopic history or any inflammatory conditions. The study was conducted in compliance with the Declaration of Helsinki and was approved by the Ethical Committee of the First Affiliated Hospital of Jinzhou Medical University (approval no.: KY201702). After obtaining written informed consent, approximately 10 mL of peripheral blood from each patient (acute attack stage) was taken into a K2EDTA‐containing tube and centrifuged at 450 *g* for 10 min. Resultant cells and plasma were collected separately for flowcytometric and ELISA analysis, respectively.

### 2.3 Flowcytometric Cell Analysis and Sorting, Cytospin Slide Preparation and Cell Counting

We followed the methods of Hua Xie et al. [26] for flowcytometric cell analysis and sorting, cytospin slide preparation and differential cell counting. Briefly, blood samples were preincubated with human Fc receptor blocking solution and Zombie Aqua dye. Cells were subsequently incubated with each fluorescein conjugated mAb, including APC‐CCR3, PerCP‐FcεRIα, PE‐CD203c and PE/Cy7‐CD63. After lysing erythrocytes and excluding dead cells and doublets, a total of 100,000 events in the live cell gate were acquired with FACSVerse flowcytometer (BD Biosciences), and analysed with FlowJo software version 7.0 (Treestar). Control samples were stained with each irrelevant isotype‐ and concentration‐matched Ab of anti‐human CCR3, CD63, CD203c and FcεRIα. The gating strategy for CD63, CD203c and FcεRIα expressions on CCR3^+^ cells of granulocytes and PBMC was demonstrated.

Fresh peripheral blood samples were preincubated with human Fc receptor blocking solution and Zombie Aqua dye, and then stained with APC‐CCR3 mAb. Erythrocytes were lysed, and CCR3^+^ granulocytes and PBMC were sorted into individual tubes using a three‐laser SH800 sorter (Sony Biotechnology) in purity mode.

Cytocentrifuge preparations were made with Shandon Cytospin 4 (ThermoFisher Scientific) and stained with Wright‐Giemsa stain. Differential cell counts were performed with a minimum of 200 cells and were expressed as relative percentages of each cell type per preparation.

### 2.4 Determination of PGD_2_ , LTC_4_ and EDN Levels in Human Plasma

Human plasma levels of PGD_2_, LTC_4_ and EDN were determined using ELISA kits according to the manufacturers' instructions. The sensitivity for PGD_2_, LTC_4_ and EDN was 117.2 pg/mL, 17.1 pg/mL and 6.25 U/mL, respectively.

### 2.5 Statistics

G*power 3.1 software was performed to assess sample size under conditions with an effect size f 0.25 (0.05 α error, 0.8 power and 5 groups) [27]. Statistical analyses were performed using SPSS27.0. Data for peripheral blood assays were displayed as scatter plots or boxplots, where Kruskal–Wallis analysis indicated significant differences between groups; the Mann–Whitney test with Bonferroni correction was employed for further pairwise comparisons [28]. Correlations were analysed using bivariate analysis with Pearson's or Spearman's *rho* (*ρ*) correlation test. Correlation coefficient ranges were defined as follows: *R* < 0.3 as a weak correlation, 0.3 ≤ *R* ≤ 0.7 as a moderate correlation and *R* > 0.7 as a strong correlation [29]. Normality of distribution was determined by Kolmogorov–Smirnov or Shapiro–Wilk test. The receiver operator characteristic (ROC) curve was determined. Area under the curve (AUC), sensitivity and specificity were calculated from the ROC curve according to the cutoff value that maximised the Youden index. Reference ranges were presented as the 2.5th and 97.5th percentile (95% confidence interval, 95% CI). For all analyses, *p* < 0.05 was considered statistically significant.

## 3 Results

### 3.1 Altered Proportions and Absolute Numbers of CCR3 ^+^ Cells in Granulocytes and PBMC of AA and ARA Patients

**FIGURE 1:**
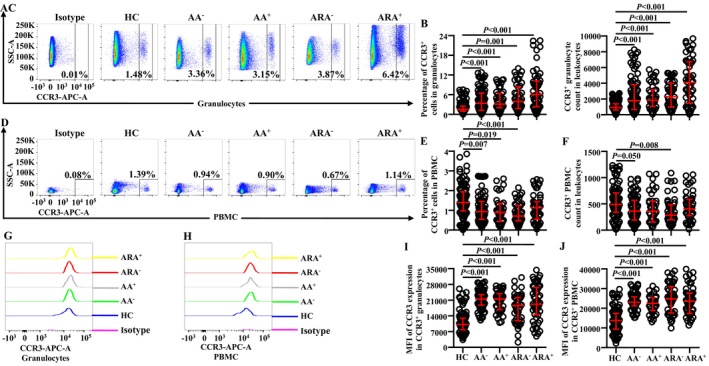
Flow cytometry analysis of proportions and absolute numbers of CCR3^+^ cells in granulocytes and PBMC of allergic asthma (AA), combined allergic rhinitis and asthma syndrome (ARA) and healthy control (HC) subjects. Percentages and absolute numbers of CCR3^+^ cells in granulocytes (A–C) and PBMC (D–F) of common airborne allergen‐negative AA (AA^−^), −positive AA (AA^+^), −negative ARA (ARA^−^), −positive ARA (ARA^+^) patients and HC subjects were shown. Representative graphs of mean fluorescent intensity (MFI) of CCR3 in CCR3^+^ granulocytes (G) and PBMC (H). MFI of CCR3 expression levels in CCR3^+^ granulocytes (I) and PBMC (J). Indicated cells were counted in 100,000 leukocytes. Data were displayed as scatter plots, which indicate the median and the interquartile range. *p* < 0.05 was taken as statistically significant.

Little is known of the changes in proportions and absolute numbers of CCR3^+^ cells in granulocytes of AA and ARA; we investigated them herein. The results showed that the percentages of CCR3^+^ cells in granulocytes (Figure 1A,B) and absolute numbers of CCR3^+^ granulocytes (Figure 1C) enhanced dramatically, and the mean fluorescent intensity (MFI) of CCR3 on CCR3^+^ granulocytes was elevated in AA and ARA patients in comparison with HC subjects (Figure 1G,I).

In order to confirm changes in the expressions of CCR3 in PBMC of AA and ARA, we examined CCR3^+^ PBMC in the present study. The results showed that the proportions of CCR3^+^ cells decreased in PBMC of AA^−^, AA,^+^ and ARA^−^ patients (Figure 1D,E). The absolute numbers of CCR3^+^ PBMC decreased in AA^−^ and ARA^−^ patients (Figure 1F). The MFI of CCR3 on CCR3^+^ PBMC enhanced in AA and ARA patients (Figure 1H,J).

### 3.2 Identification of Isolated CCR3 ^+^ Cells in Granulocytes and PBMC of AA and ARA Patients

**FIGURE 2:**
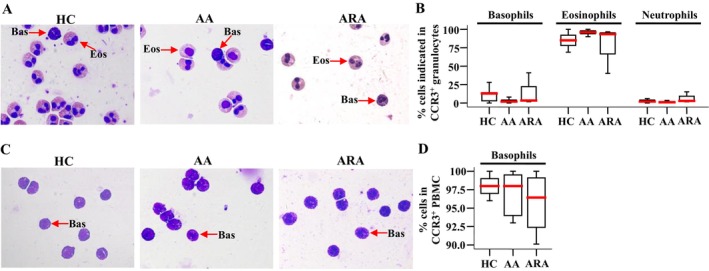
Differential identification of isolated CCR3^+^ cells from human peripheral blood. Representative images of isolated CCR3^+^ granulocytes (A) and PBMC (C), and percentages of cells indicated in CCR3^+^ granulocytes (B) and PBMC (D). Data were displayed as boxplots, which indicate the median, the interquartile range, the largest value and the smallest value. Each data represented a group of 5–7 separate experiments. Magnification was ×100. *p* < 0.05 was taken as statistically significant.

In order to confirm the cell types that express CCR3 in peripheral blood granulocytes, we isolated CCR3^+^ cells from human peripheral blood and examined them under the microscope. The results showed that up to 96.0% of CCR3^+^ cells in granulocytes were eosinophils, and basophils consisted of up to 12.8% in CCR3^+^ granulocytes (Figure 2A,B). We confirmed that more than 90.1% of CCR3^+^ cells in PBMC were basophils (Figure 2C,D).

### 3.3 Altered Expressions of CD63 in CCR3 ^+^ Granulocytes and PBMC of AA and ARA Patients

**FIGURE 3:**
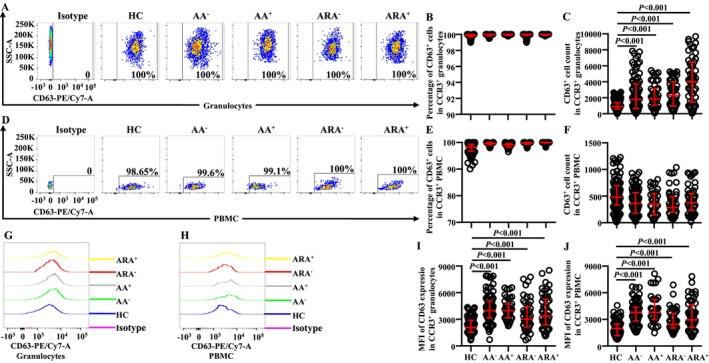
Flow cytometry analysis of CD63 expression in CCR3^+^ cells of allergic asthma (AA), combined allergic rhinitis and asthma syndrome (ARA) and healthy control (HC) subjects. Percentages and absolute numbers of CD63^+^CCR3^+^ cells in granulocytes (A–C) and PBMC (D–F) of common airborne allergen‐negative AA (AA^−^), −positive AA (AA^+^), −negative ARA (ARA^−^), −positive ARA (ARA^+^) patients and HC subjects were shown. Representative graphs of mean fluorescent intensity (MFI) of CD63 in CCR3^+^granulocytes (G) and PBMC (H). MFI of CD63 expression levels in CCR3^+^ granulocytes (I) and PBMC (J). Indicated cells were counted in 100,000 leukocytes. Data were displayed as scatter plots, which indicate the median and the interquartile range. *p* < 0.05 was taken as statistically significant.

CD63 has been considered as an activation marker of basophils [9]. However, little is known of the expression of CD63 on CCR3^+^ granulocytes of AA and ARA. We found that almost all CCR3^+^ cells expressed CD63 regardless of whether they were in granulocyte (Figure 3A,B) or PBMC populations (Figure 3D,E) of HC, AA and ARA subjects. The numbers of CD63^+^CCR3^+^ granulocytes of AA and ARA patients increased (Figure 3C). MFI of CD63 in CCR3^+^ granulocytes enhanced in AA and ARA patients (Figure 3G,I).

The numbers of CD63^+^CCR3^+^ PBMC seemed to decrease in AA and ARA patients but without statistical significance (Figure 3F). MFI of CD63 in CCR3^+^ PBMC markedly enhanced in AA and ARA patients (Figure 3H,J).

### 3.4 Altered Expressions of CD203c on CCR3 ^+^ Granulocytes and PBMC of AA and ARA Patients

**FIGURE 4:**
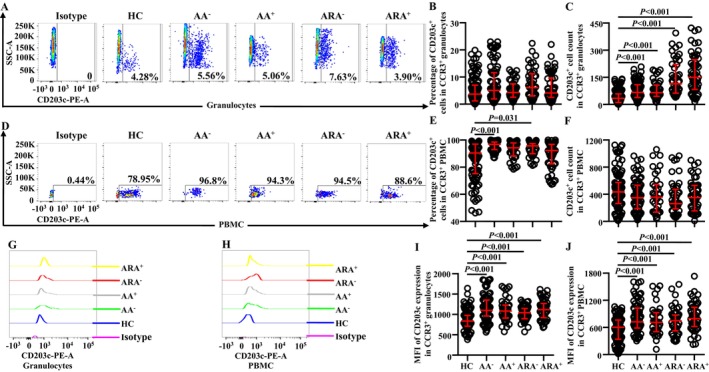
Flow cytometry analysis of CD203c expression in CCR3^+^ cells of allergic asthma (AA), combined allergic rhinitis and asthma syndrome (ARA) and healthy control (HC) subjects. Percentages and absolute numbers of CD203c^+^CCR3^+^ cells in granulocytes (A–C) and PBMC (D–F) of common airborne allergen‐negative AA (AA^−^), −positive AA (AA^+^), −negative ARA (ARA^−^), −positive ARA (ARA^+^) patients and HC subjects were shown. Representative graphs of mean fluorescent intensity (MFI) ofCD203c in CCR3^+^ granulocytes (G) and PBMC (H). MFI of CD203c expression levels in CCR3^+^ granulocytes (I) and PBMC (J). Indicated cells were counted in 100,000 leukocytes. Data were displayed as scatter plots, which indicate the median and the interquartile range. *p* < 0.05 was taken as statistically significant.

Increased CD203c^+^ basophils were observed in the blood from patients with asthma exacerbation [14]. However, little is known about the expression of CD203c on CCR3^+^ granulocytes. We found that the numbers (Figure 4C) but not the percentages (Figure 4A,B) of CD203c^+^ cells markedly increased in CCR3^+^ granulocytes of AA and ARA patients. MFI of CD203c on CCR3^+^ granulocytes also greatly elevated in AA and ARA patients (Figure 4G,I).

Regarding PBMC population, the percentages of CD203c^+^ cells out of CCR3^+^ PBMC of AA^−^ and ARA^−^ patients clearly enhanced (Figure 4D,E), whereas the number of CCR3^+^CD203c^+^ PBMC between groups showed no statistically significant changes (Figure 4F). MFI of CD203c on CCR3^+^ PBMC of AA and ARA patients dramatically enhanced (Figure 4H,J).

### 3.5 Altered Expressions of FcεRIα on CCR3 ^+^ Granulocytes and PBMC of AA and ARA Patients

**FIGURE 5:**
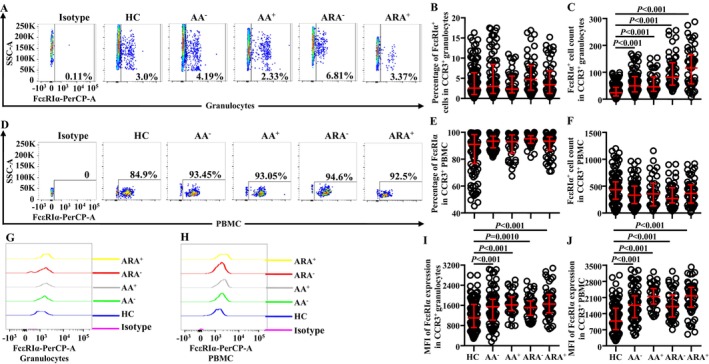
Flow cytometry analysis of FcεRIα expression in CCR3^+^ cells of allergic asthma (AA), combined allergic rhinitis and asthma syndrome (ARA) and healthy control (HC) subjects. Percentages and absolute numbers of FcεRIα^+^CCR3^+^ cells in granulocytes (A–C) and PBMC (D–F) of common airborne allergen‐negative AA (AA^−^), −positive AA (AA^+^), −negative ARA (ARA^−^), −positive ARA (ARA^+^) patients and HC subjects were shown. Representative graphs of mean fluorescent intensity (MFI) of FcεRIα in CCR3^+^granulocytes (G) and PBMC (H). MFI of FcεRIα expression levels in CCR3^+^ granulocytes (I) and PBMC (J). Indicated cells were counted in 100,000 leukocytes. Data were displayed as scatter plots, which indicate the median and the interquartile range. *p* < 0.05 was taken as statistically significant.

FcεRIα has long been accepted as a receptor of IgE on basophils, which mediates allergen‐induced basophil activation [26]. We therefore investigated FcεRIα expression on CCR3^+^ cells. The results showed that the numbers (Figure 5C) but not the proportions (Figure 5A,B) of FcεRIα^+^ cells in CCR3^+^ granulocytes of AA and ARA patients increased. MFI of FcεRIα on CCR3^+^ granulocytes of AA and ARA patients markedly enhanced (Figure 5G,I).

In PBMC, however, neither the numbers (Figure 5F) nor the proportions (Figure 5D,E) of FcεRIα^+^ cells in CCR3^+^ PBMC of AA and ARA patients changed. MFI of FcεRIα on CCR3^+^ PBMC of AA and ARA patients dramatically increased (Figure 5H,J).

### 3.6 Correlations Between CD63, CD203c and FcεRIα Expression in Granulocytes and PBMC of AA and ARA Patients

**FIGURE 6:**
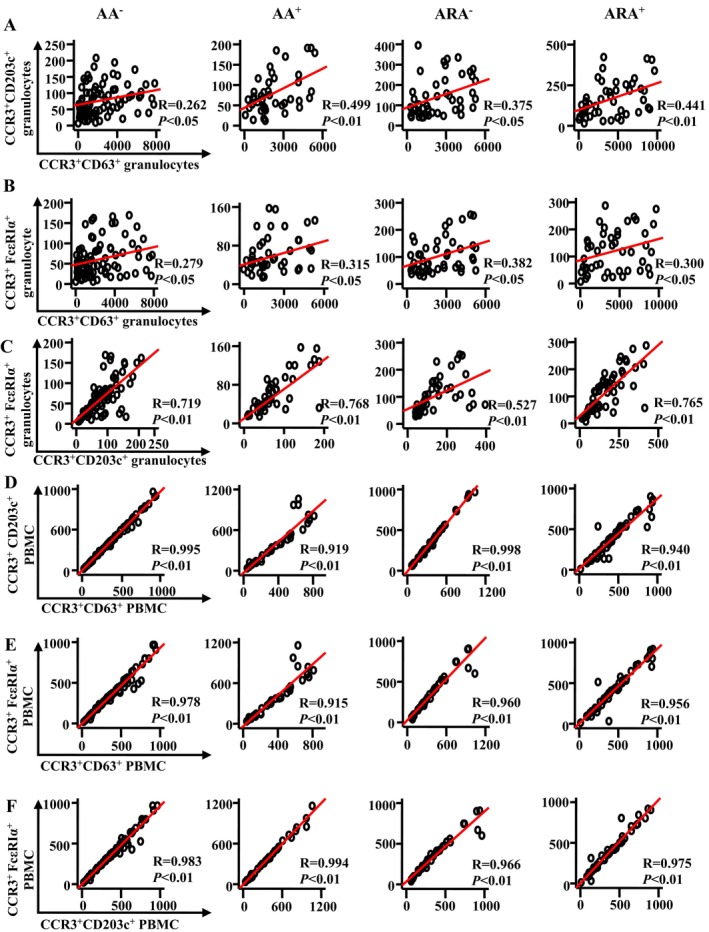
Scatter plots, linear regression lines and coefficients of determinations between the variables of patients with asthma (AA), combined allergic rhinitis and asthma syndrome (ARA) and healthy control (HC) subjects. Correlations between absolute numbers of CD63^+^, CD203c^+^ and FcεRIα^+^ cells in CCR3^+^ granulocytes (A–C) and PBMC (D–F) of common airborne allergen‐negative AA (AA^−^), −positive AA (AA^+^), −negative ARA (ARA^−^), −positive ARA (ARA^+^) patients and HC subjects were analysed using Pearson's correlation test. Indicated cells were counted in 100,000 leukocytes. Correlation coefficient ranges were defined as follows: *R* < 0.3 as a weak correlation, 0.3 ≤ *R* ≤ 0.7 as a moderate correlation, and *R* > 0.7 as a strong correlation. *p* < 0.05 was taken as statistically significant.

In order to learn more about the relationships between expressions of CD63, CD203c and FcεRIα in CCR3^+^ granulocytes and PBMC of AA and ARA patients, Pearson's correlation test was employed. It was observed that there were moderate correlations between the numbers of CD63^+^CCR3^+^ and CD203c^+^CCR3^+^ granulocytes (Figure 6A) and between the numbers of CD63^+^CCR3^+^ and FcεRIα^+^CCR3^+^ granulocytes (Figure 6B) of AA^+^ and ARA patients, a weak correlation between the numbers of CD63^+^CCR3^+^ and CD203c^+^CCR3^+^ granulocytes (Figure 6A) and between the numbers of CD63^+^CCR3^+^ and FcεRIα^+^CCR3^+^ granulocytes (Figure 6B) of AA^−^ patients, and relatively strong correlations between the numbers of CD203c^+^CCR3^+^ and FcεRIα^+^CCR3^+^ granulocytes of AA and ARA patients (Figure 6C).

On the other hand, strong correlations between the cell numbers of CD63^+^CCR3^+^, CD203c^+^CCR3^+^ and FcεRIα^+^CCR3^+^ PBMC of AA and ARA patients were achieved (Figure 6D–F).

### 3.7 Elevated Plasma Levels of EDN, LTC_4_ and PGD_2_ in AA and ARA Patients

**FIGURE 7:**
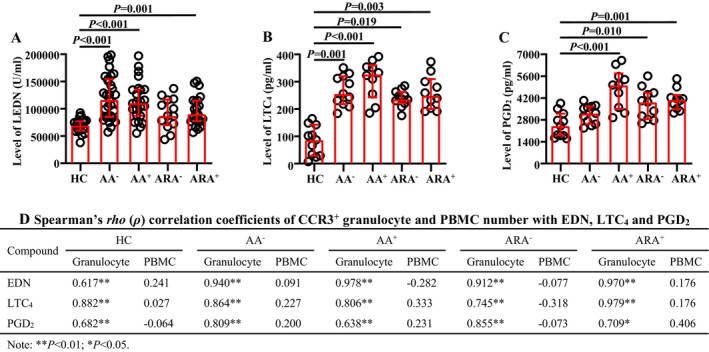
ELISA analysis of plasma levels of EDN, LTC_4_ and PGD_2_ in allergic asthma (AA), combined allergic rhinitis and asthma syndrome (ARA) and healthy control (HC) subjects. Plasma levels of EDN (A), LTC_4_ (B) and PGD_2_ (C) in common airborne allergen‐negative AA (AA^−^), −positive AA (AA^+^), −negative ARA (ARA^−^), −positive ARA (ARA^+^) patients and HC subjects, and their Spearman's *rho* (*ρ*) correlation coefficients with the absolute numbers of CCR3^+^ granulocytes and PBMC (D). Correlation coefficient ranges were defined as follows: *R* < 0.3 as a weak correlation, 0.3 ≤ *R* ≤ 0.7 as a moderate correlation and *R* > 0.7 as a strong correlation. p < 0.05 was taken as statistically significant.

EDN, LTC_4_ and PGD_2_ have been considered to have potential diagnostic value in AA [23, 30, 31]. To distinguish basophil degranulation from eosinophil degranulation based on their cell sources, we investigated the plasma levels of EDN, LTC_4_ and PGD_2_ in AA and ARA patients and explored their correlations with CCR3^+^ granulocytes and PBMC. The results showed that the plasma levels of EDN in AA and ARA^+^, LTC_4_ in AA and ARA, and PGD_2_ in AA^+^ and ARA patients markedly increased (Figure 7A–C).

Additionally, moderate to strong correlations were shown in terms of CCR3^+^ granulocyte but not PBMC numbers with the plasma levels of EDN, LTC_4_ and PGD_2_ in HC, AA and ARA subjects (Figure 7D).

### 3.8 The Diagnosis Performance of AA and ARA Patients Based on CCR3 ^+^ CD203c ^+^ Granulocyte Numbers

**FIGURE 8:**
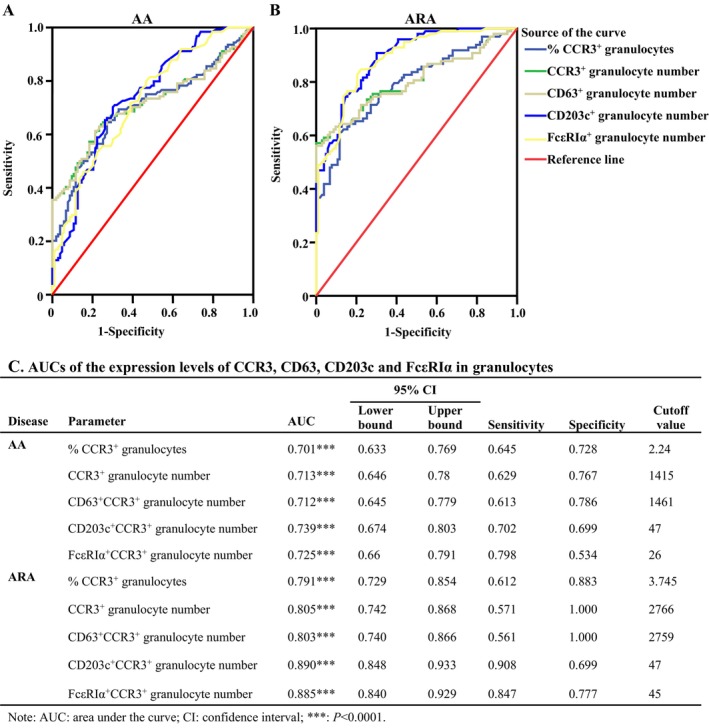
AUCs of the expression levels of CCR3, CD63, CD203c and FcεRIα in granulocytes of patients with asthma (AA) and combined allergic rhinitis and asthma syndrome (ARA). The receiver operator characteristic (ROC) curve for assessing the diagnostic performance of the percentages of CCR3^+^ granulocytes, and absolute numbers of CCR3^+^, CCR3^+^CD63^+^, CCR3^+^CD203c^+^ and FcεRIα^+^CCR3^+^ granulocytes in patients with AA (A) and ARA (B). Area under the curve (AUC), sensitivity and specificity of the expression levels of CCR3, CD63, CD203c and FcεRIα in granulocytes (C). AUC, sensitivity and specificity were calculated from the ROC curve according to the cutoff value that maximised the Youden index. Reference ranges were presented as the 2.5th and 97.5th percentiles (95% confidence interval, 95% CI). *p* < 0.05 was taken as statistically significant.

We then further assess the diagnostic performance of CCR3^+^ and CCR3^+^CD203c^+^ granulocytes of AA and ARA patients. As shown in Figure 8A–C, all the parameters in granulocytes, including the percentages and the numbers of CCR3^+^ granulocytes, as well as CD63^+^, CD203c,^+^ and FcεRIα^+^ cell numbers in CCR3^+^ granulocytes, seemed to have diagnostic value for AA and ARA, which yielded AUC values of more than 0.7 and 0.8 for AA and ARA, respectively (Figure 8C). To be noted, the Yoden index achieved the highest values for CD203c^+^CCR3^+^ granulocyte numbers, with a sensitivity of 0.702 and 0.908 and a specificity of 0.699 and 0.699 for AA and ARA patients, respectively (Figure 8C).

## 4 Discussion

One of the most striking findings in the present study is that both the percentages and the absolute numbers of CCR3^+^ cells, and the density of CCR3 expression on a single cell were dramatically enhanced in the granulocytes of AA and ARA patients. As CCR3 is a receptor for eotaxins, RANTES, the monocyte chemotactic proteins, and plays a major role in allergic diseases [32], upregulated CCR3 expression is likely to participate in the development of AA and ARA, and could be a marker for the auxiliary diagnosis of AA and ARA.

On the other hand, the proportion of CCR3^+^ PBMC decreased in peripheral blood of AA and ARA^−^ patients in the current study, which is an unexpected result since enhanced expression of CCR3^+^ basophils has been noticed in allergic conditions such as food [33], grass pollen [11] and drug allergy [34]. A report that a decreased percentage of CD123^+^ basophils was observed in the blood after allergen challenge of asthmatic lung may help explain our observation because basophils might be directly recruited from the blood circulation to the airway lumen [35]. As CCR3 is a stable and highly expressed basophils selection marker, which allows accurate identification of basophils in PBMC without the need for a second marker [12], the reduced number of CCR3^+^ PBMC in AA and ARA^−^ patients may implicate the infiltration of basophils to the asthmatic lung.

In contrast to the report that CCR3 is a stable basophil selection marker in PBMC, independent of atopic background or basophil activation state [36], we observed that the density of CCR3 expression on a single cell was dramatically enhanced in both granulocytes and PBMC of AA and ARA patients. This result indicates that CCR3^+^ cells may be actively involved in AA and ARA and that the MFI of CCR3 expression could aid AA and ARA diagnosis.

In order to confirm the cell types that express CCR3 in granulocytes and PBMC, we examined isolated blood CCR3^+^ cells under the microscope. As almost all CCR3^+^ PBMC are basophils, changes in the proportions, numbers and MFI of these cells confidently represent basophils. As CCR3^+^ granulocytes are mainly eosinophils, and basophils consist of only a small proportion of them, upregulated expression of CCR3^+^ granulocytes should represent more eosinophils than basophils in the granulocyte population. The reports that upregulated expression of CCR3 on eosinophils are associated with asthma [8] and that the number of activated basophils is increased in the sputum of patients with eosinophilic asthma [36] may support the notion that CCR3^+^ granulocytes are involved in AA and ARA.

CD203c and CD63 have been recognised as activation markers of basophils. It appears that CD203c is a better marker than CD63 for the identification of basophils. For example, it is reported that CD203c upregulation occurs in the whole basophil population, whereas CD63 expression occurs only in a minority of basophils [37]. Upregulation of CD203c proved to be a more consistent activation marker than CD63 [38], and increased expression of CD203c but not CD63 on basophils is accompanied by asthma exacerbation [14]. We find that almost all CCR3^+^ cells expressed CD63 regardless of whether they were in granulocyte or PBMC populations of HC, AA or ARA subjects. The reports that peripheral blood eosinophils constitutively express CD63 [39, 40], and that up to 100% unstimulated basophils express CD63 though with large variation [26, 41, 42, 43] seem to support our observation. The enhanced MFI expressions of CD63 in CCR3^+^ granulocytes and PBMC in the present study implicate that these cells, possibly including basophils, eosinophils and neutrophils, are activated in AA and ARA. We also find almost all basophils are CD203c positive in both HC and diseased groups in the current study, which seems to be validated by our previous studies [26, 41] and the study of Zehwan Kim et al. [44]. Similarly, increased CD203c expression on granulocytes and PBMC indicates that activated basophils are elevated in AA and ARA patients because CD203c is a selective marker of basophil activation [11, 44]. Based on these results, we believe that both CD63 and CD203c are sensitive enough as activation markers, but CD203c seems more selective for basophils.

Similarly to CD63 and CD203c, enhanced MFI expression of FcεRIα^+^ cells is found in CCR3^+^ granulocytes and PBMC. As FcεRIα expression has been observed in basophils [45] and eosinophils [46], and FcεRIα plays a central role in the initiation and control of allergic inflammation, which can mediate allergen‐induced basophil activation [47], the increased number of FcεRIα^+^ cells most likely belongs to activated basophils and eosinophils in AA and ARA.

Furthermore, the differential CCR3^+^ cell types in granulocytes and PBMC may explain well the differential correlation degrees between the numbers of CD63^+^CCR3^+^, CD203c^+^CCR3,^+^ and FcεRIα^+^CCR3^+^ granulocytes and PBMC of HC, AA, and ARA subjects in the current study.

Notably, elevated plasma levels of EDN, LTC_4_ and PGD_2_ in AA and ARA patients are moderately to highly correlated with CCR3^+^ granulocyte but not PBMC numbers in our current study. As EDN is one major protein of eosinophil granules and is produced by eosinophils but not basophils [22], and LTC_4_ and PGD_2_ are produced mainly by eosinophils and basophils [17, 18, 48] these implicate that CCR3^+^ granulocytes, which primarily include eosinophils, are largely responsible for the elevated EDN, LTC_4_ and PGD_2_ in AA and ARA patients.

As blood basophils [49] and eosinophils [10] express high levels of CCR3, and basophils but not eosinophils constitutively express CD203c [50], we believe that CCR3^+^CD203c^+^ cells represent basophils, and CCR3^+^CD203c^−^ cells represent eosinophils in granulocytes. As both basophils and eosinophils are classical effector cells for AA and allergic rhinitis [51], AA and allergic rhinitis are united airway diseases [52], a combination of CCR3 and CD203c should be a reliable diagnostic test for AA and ARA, which is validated further by ROC curve analysis in the current study.

## 5 Conclusion

In conclusion, discovering granulocyte basophils in peripheral blood has provided greater scope for basophil research and the clinical use of basophil tests, even though CCR3^+^ cells do not solely represent basophils in this cell population. A combination of CCR3 and CD203c is likely a reliable test for the diagnosis of AA and ARA since they represent basophils in PBMC and granulocytes and eosinophils in granulocytes, and the expressions of CCR3 and CD203c are clearly enhanced in the granulocytes of AA and ARA.

## Author Contributions

**Junling Wang:** conceptualization (equal), formal analysis (lead), funding acquisition (equal), investigation (lead), methodology (lead), resources (equal), software (lead), validation (lead), visualization (lead), writing – original draft (equal), writing – review and editing (equal). **Mengmeng Zhan:** formal analysis (equal), investigation (equal), methodology (equal), resources (equal), software (equal), supervision (equal), validation (equal), visualization (equal), writing – review and editing (equal). **Fangqiu Gu:** data curation (equal), formal analysis (equal), methodology (equal), software (equal), validation (equal), visualization (equal), writing – review and editing (equal). **Huiyun Zhang:** funding acquisition (equal), project administration (equal), resources (equal), supervision (equal), writing – review and editing (equal). **Hua Xie:** conceptualization (equal), project administration (equal), resources (equal), writing – review and editing (equal). **Bingyu Qin:** project administration (equal), resources (equal), supervision (equal), writing – review and editing (equal). **Shaoheng He:** conceptualization (lead), data curation (lead), funding acquisition (equal), methodology (lead), project administration (lead), resources (equal), supervision (lead), writing – review and editing (lead).

## Ethics Statement

Research ethical approval was obtained from the Ethical Committee of the First Affiliated Hospital of Jinzhou Medical University (KY201702).

## Consent

Informed consent was obtained from all subjects involved in the study. Written informed consent was obtained from all subjects to publish this paper.

## Conflicts of Interest

The authors declare no conflicts of interest.

## Supporting information


**Figure S1.** Gating strategies for CCR3^+^ cells in peripheral blood granulocyte and mononuclear cell (PBMC) population, and for CD63, CD203c and FcεRIα expressions on CCR3^+^ cells.
**Table S1**. General characteristics of volunteers.

## Data Availability

Data that support the findings of this study are available from the corresponding author upon reasonable request.
