# Supplementary material for: Diagnostic Use of CCR3, CD63, CD203c and FcεRIα on Blood Leukocytes of Allergic Asthma and Combined Allergic Rhinitis and Asthma Syndrome
Source: J Cell Mol Med. 2025 Jun 18;29(12):e70594. doi: 10.1111/jcmm.70594 (PMC12176697; doi:10.1111/jcmm.70594)
Supplement: Supplementary file 1 — Figure S1. Gating strategies for CCR3+ cells in peripheral blood granulocyte and mononuclear cell (PBMC) population, and for CD63, CD203c and FcεRIα expressions on CCR3+ cells. Table S1. General characteristics of volunteers. [file JCMM-29-e70594-s001.docx]

**Supplement Table 1** General characteristics of volunteers.

| Population | Case | Age (y) | Female/male | History (y) | Onset age (y) |
| --- | --- | --- | --- | --- | --- |
| HC | 103 | 30 (12-81) | 61/42 | 0 | NA |
| AA^-^ | 82 | 48 (6-75) | 54/28 | 3 (0.25-30) | 38 (1-74.75) |
| AA^+^ | 43 | 43 (7-74) | 24/19 | 4 (0.25-30) | 34 (2-70) |
| ARA^-^ | 46 | 41 (8-70) | 29/17 | 3 (0.5-29) | 35.5 (6-58) |
| ARA^+^ | 52 | 34 (5-63) | 28/24 | 4.5 (0.5-33) | 29 (3-55) |

Notes: Median values (range) are shown. Common airborne allergens (house dust mite, European house dust mite, house dust, mugwort, Sieversian wormwood, short ragweed, Giant ragweed, hop, rice, wheat, maize, plane tree, pine, cypress, common silver birch, olive, black mould, Alternaria plant rot fungus, German cockroach, American cockroach, cat and dog) were examined by skin prick test. Serum airborne allergen-sIgE (house dust, tree pollen mix, weed pollen mix, animal protein mix, mould mix, house dust mite, German cockroach, common silver birch, common ragweed, mugwort, cocklebur, white goosefoot, cat dander and common mold) and food allergen-sIgE (egg white, cow’s milk, peanut, soybean, crab, shrimp, sea fish mix and fresh water fish mix) were examined using ImmunoCAP (Phadia 100, Thermo). AA^-^ = asthma for negative common airborne allergen test; AA^+^ = asthma for positive common airborne allergen test; ARA^-^ = combined allergic rhinitis and asthma syndrome for negative common airborne allergen test; ARA^+^ = combined allergic rhinitis and asthma syndrome for positive common airborne allergen test.

**Supplement Figure 1**

**FSC-H**

**50K**

**250K**

**100K**

**150K**

**200K**

**0**

**FSC-A**

**0**

**50K**

**150K**

**250K**

**CCR3-APC-A**

**0**

**10^3^**

**10^5^**

**10^4^**

**-10^3^**


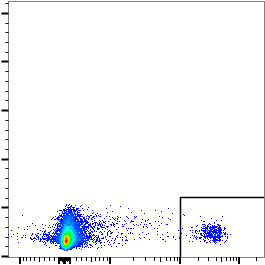

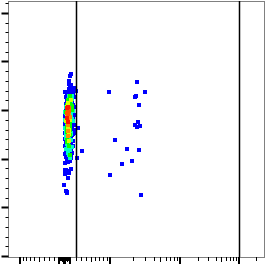

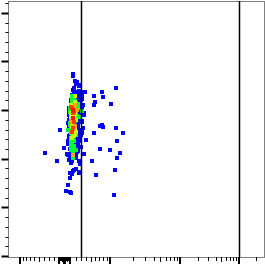

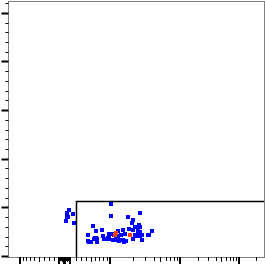

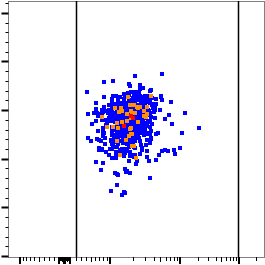

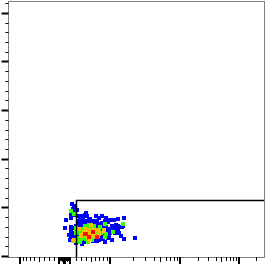

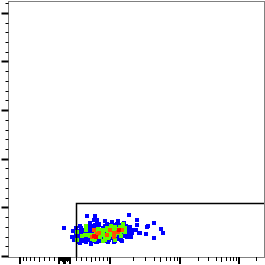


**Granulocytes**

**PBMC**

**SSC-A**

**50K**

**250K**

**100K**

**150K**

**200K**

**0**

**FSC-A**

**0**

**50K**

**150K**

**250K**

**SSC-A**

**100K**

**50K**

**250K**

**150K**

**200K**

**0**

**50K**

**250K**

**100K**

**150K**

**200K**

**0**

**SSC-A**

**0**

**10^3^**

**10^5^**

**10^4^**

**-10^3^**

**CCR3-APC-A**

**CD63-PE/Cy7-A**

**SSC-A**

**-10^3^**

**0**

**10^3^**

**10^5^**

**10^4^**

**50K**

**250K**

**100K**

**150K**

**200K**

**0**

**SSC-A**

**CD63-PE/Cy7-A**

**-10^3^**

**0**

**10^3^**

**10^5^**

**10^4^**

**50K**

**250K**

**100K**

**150K**

**200K**

**0**

**CD203c-PE-A**

**-10^3^**

**0**

**10^3^**

**10^5^**

**10^4^**

**50K**

**250K**

**100K**

**150K**

**200K**

**0**

**CD203c-PE-A**

**SSC-A**

**-10^3^**

**0**

**10^3^**

**10^5^**

**10^4^**

**50K**

**250K**

**100K**

**150K**

**200K**

**0**

**FcεRIα-PerCP-A**

**SSC-A**

**-10^3^**

**0**

**10^3^**

**10^5^**

**10^4^**

**50K**

**250K**

**100K**

**150K**

**200K**

**0**

**FcεRIα-PerCP-A**

**SSC-A**

**-10^3^**

**0**

**10^3^**

**10^5^**

**10^4^**

**50K**

**250K**

**100K**

**150K**

**200K**

**0**

**SSC-A**

**100%**

**10.1%**

**3.22%**

**93.1%**

**90.1%**

**98.3%**


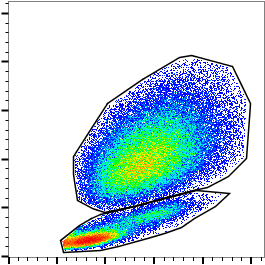

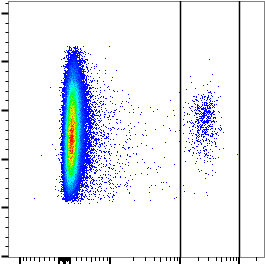

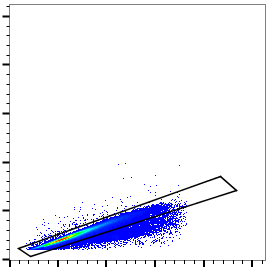


**SSC-A**

**50K**

**250K**

**100K**

**150K**

**200K**

**0**

**Zombie Aqua-A**

**-10^3^**

**0**

**10^3^**

**10^4^**

**10^5^**


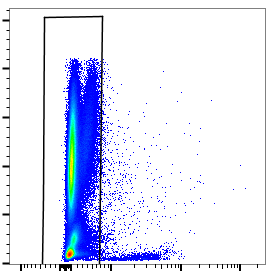


**Supplement Fig. 1 Gating strategies for CCR3^+^ cells in granulocyte and peripheral blood mononuclear cell (PBMC) population, and for CD63, CD203c and FcεRIα expressions on CCR3^+^ cells.**
